# Description and molecular data of a new cestode parasite, *Cladotaenia anomala* n. sp. (Paruterinidae) from the Australasian harrier (*Circus approximans* Peale) in New Zealand

**DOI:** 10.1007/s11230-024-10147-2

**Published:** 2024-03-06

**Authors:** Bronwen Presswell, Jerusha Bennett

**Affiliations:** Department of Zoology, University of Otago, PO Box 56, Dunedin, New Zealand

## Abstract

Currently comprising 12 species infecting the gastrointestinal tracts of diurnal raptors (Falconiformes, Accipitriformes), species of *Cladotaenia* are diagnosed by their branching uterus, testes in two fields reaching the same level anteriorly, and small rostellum armed with taenioid hooks arranged in two rows. In this study we describe a new species of *Cladotaenia* recovered from a number of Australasian harriers *Circus approximans,* from the southern half of South Island, New Zealand. The new species is distinguished from other species by its single circle of hooks. It is closest, morphologically, to *C. circi*, but differs in the shape of the terminal proglottids and the number of uterine branches. Sequences of 28S and *cox1* gene are presented. Genetically, *Cladotaenia anomala* **n. sp.** is closest to *Cladotaenia globifera* but differs morphologically in the size of the suckers, testes and eggs. This description constitutes the first record of a *Cladotaenia* species in New Zealand. We discuss some potential routes this parasite may have taken to arrive in New Zealand.

## Introduction

The genus *Cladotaenia* Cohn, 1901 (Cyclophyllidea: Paruterinidae) was erected for *Taenia globifera* Batsch, 1786 which was described from hawks (Aves: Accipitridae). The genus was originally included in the family Taeniidae Ludwig, 1886 (Joyeux & Baer 1961; Abuladze 1958; Yamaguti 1959), but was believed to belong to Dilepididae Fuhrmann, 1907 by Fuhrmann & Baer (1943) a placement followed by both Freeman (1959) and Schmidt (1986). Subsequently, *Cladotaenia* was excluded from the Taeniidae on zoogeographical, morphological and ontogenetic grounds by Rausch (1985) a decision recently supported by genetic data (Guo et al. 2019). The genus is now considered a member of the family Paruterinidae (Georgiev and Kornyushin 1994, Mariaux et al. 2017), and it is a close sister taxon to *Paruterina*, the type genus of the family. In addition, the mitochondrial gene order is the same as *Paruterina* but different from members of Taeniidae (Guo et al. 2019). Genus *Paracladotaenia* Yamaguti, 1935 was also established for a cestode from a hawk, and was distinguished from *Cladotaenia* mainly by the absence of rostellar hooks. However, *Cladotaenia* spp. characteristically lose their hooks if specimens are not fixed immediately after the death of their host; consequently, Schmelz (1941) placed *Paracladotaenia* in synonymy with *Cladotaenia*, a position now widely accepted (Yamaguti 1959; Georgiev & Kornyushin 1994). Freeman (1959) reviewed in detail the complex history of the species of this genus, as well as elucidating the life cycle and providing a character for differentiating between *Cladotaenia* and *Paruterina* plerocercoids in the liver and mesenteries of small mammals. *Cladotaenia* currently contains 12 nominal species, all of which infect the gastrointestinal tract of diurnal raptors (Accipitriformes and Falconiformes), with rodents and insectivores as intermediate hosts (Georgiev & Kornyushin 1994). Species of *Cladotaenia* have been reported from Europe, Africa, East Asia, India and North America (Freeman 1959; Georgiev & Kornyushin 1994). There are specimens of *Cladotaenia* sp. from Australia in the South Australia Museum, and *C. circi* from Vanuatu in Australian and British collections. However, there are no previous records of any species of *Cladotaenia* from New Zealand.

The Australasian harrier *Circus approximans* Peale (Accipitriformes: Accipitridae), also known as swamp harrier, harrier hawk or kāhu, is native to Australia, New Zealand and some islands in the South Pacific (Debus & Kirwan 2020). The Australasian harrier, and the rare New Zealand falcon *Falco novaeseelandiae* Gmelin (Falconiformes: Falconidae), are the only two diurnal raptors extant in New Zealand. An opportunistic hunter of live prey such as small birds, mammals and invertebrates, the Australasian harrier is also a scavenger, with carrion making up a major part of the diet (Baker-Gabb 1981). In New Zealand a constant supply of road-kill carcasses has enabled the harrier to rise to very healthy population numbers (Eakle 2008). Its conservation status is Non-Threatened (Robertson et al. 2021), but the bird is considered a “taonga” (treasured) species by Māori and is partially protected by law (Wildlife (Australasian Harrier) Notice 2012). Harriers are seen frequently throughout New Zealand and are instantly recognisable. Many are themselves victims of roadkill or injury (Sadleir & Linklater 2016), and there are large numbers of deceased birds available, so it is testament to the lack of study on New Zealand wildlife parasites that not a single cestode has ever been reported for the harrier in New Zealand. Access to a number of harrier carcasses from the southern half of South Island since 2017 has allowed the authors to conduct a survey of all helminth parasites found, and what follows is a description of the paruterinid cestode *Cladotaenia* found in some of these host birds, which was found to be new to science. We provide DNA sequences of the *cox1* and 28S gene which confirm placement within Paruterinidae, and show that the new species is closest to *C. globifera* of those sequences available. A description of a new species of polymorphid acanthocephalan and a report on other helminths recovered from the New Zealand harriers, including a new species of nematode, have been published elsewhere (Presswell & Bennett 2023, 2024).

## Materials and methods

### Harrier collection and processing

In total, 65 harriers were examined for parasitic helminths: 46 individuals from Otago were donated by the Dunedin Wildlife Hospital or collected as roadkill by the first author between 2017 and 2022, and 19 individuals from Canterbury were donated by the New Zealand Raptor Trust between 2022 and 2023. Birds were frozen upon collection and defrosted prior to dissection. Cestodes were collected and preserved in 70% ethanol for whole-mount, 96% ethanol for genetic analysis and 4% buffered formalin for SEM imaging.

### Morphological data

Cestode specimens were stained using acetic iron carmine, dehydrated in an ethanol series, cleared in clove oil and mounted in Canada Balsam. Measurements were made using ImageJ software (Wayne Rasband, NIH, USA) from photographs taken on an Olympus BX51 compound microscope mounted with DP25 camera attachment (Olympus, Tokyo). All measurements are in micrometres unless otherwise indicated, and in the description are given as range, followed by mean in parentheses, where numbers permit. Drawings were made by hand from photographic series using a light box.

Specimens chosen for scanning electron microscopy (SEM) were transferred to 2.5 % gluteraldehyde in 0.1 M phosphate buffer, post-fixed in 1% osmium tetroxide and dehydrated through a gradient series of ethanols, critical-point dried in a CPD030 BalTec critical-point dryer (BalTec AG, Balzers, Liechtenstein) using carbon dioxide, mounted on aluminium stubs, and sputter coated with gold/palladium (60:40) to a thickness of 10 nm in an Emitech K575X Peltier-cooled high-resolution sputter coater (EM Technologies, Ashford, Kent, UK). The specimens were viewed with a JEOL 6700 F field emission scanning electron microscope (JEOL Ltd., Tokyo, Japan) at the Otago Centre for Electron Microscopy (OCEM, University of Otago, New Zealand).

### Molecular data and genetic distances

Nine specimens were chosen for DNA sequencing. Genomic DNA was extracted using the DNeasy® Blood & Tissue Kit (Qiagen, Hilden, Germany) according to the manufacturer’s protocol. A partial fragment of 28S rRNA gene was amplified using T16 and T30 primers (Harper & Saunders, 2001) and conditions of Bennett et al. (2023). Additionally, a partial fragment of *cox1* mitochondrial gene was amplified, using primers JB3 (Bowles et al. 1992) and trem.cox.rrnl (Králová-Hromadová et al. 2001) and conditions following Bennett and Presswell (2019). PCR products were cleaned using EXOSAP Express PCR Product Cleanup Reagent (USB Corporation, Cleveland, OH, USA), following manufacturer’s instructions. Sanger sequencing by capillary electrophoresis was performed by the Genetic Analysis Service, Department of Anatomy, University of Otago (Dunedin, New Zealand). Successfully amplified sequences were imported to Geneious Prime®v1.2, trimmed using the trim function with default parameters, and manually edited for incorrect or ambiguous base calls. A contiguous sequence was assembled for each sequence and an alignment with one representative of each unique *cox*1 haplotype was created with closely related species within Family Paruterinidae found in a NCBI Blast search. Uncorrected pairwise genetic divergences were calculated in MEGA v.11.

### Parasitological indices

We compared infection parameters of the birds examined here with those of existing *Cladotaenia* infections from accipitriform hosts where at least 30 host individuals were reported (See Table 1). The infection parameters presented include the number of *Cladotaenia* specimens recovered from all hosts, range, mean or maximum intensity as given in the source, and prevalence.

**Table 1:** Prevalence and intensity data reported for species of *Cladotaenia* in the literature. Includes only those records where the number of birds examined was greater than 30 (N). Av. = average and Max. = maximum intensity

Species	Host	N	Preval-ence N	Prevalence percent	Intensity	Locality	Reference
*C. anomala* **n. sp.**	*Circus approximans*	65	37	57%	1 to15+	New Zealand	This study
*C. globifera*	*Falco tinnunculus*	73	3	4.1%	1 to 2	Slovak Republic	Komorová et al. 2017
*C. globifera*	*Buteo buteo*	119	43	36.1%	1 to 20	Slovak Republic	Komorová et al. 2017
*C. globifera*	*Buteo buteo*	84	31	37%	Max. 18	Germany	Krone 2000
*C. globifera*	*Buteo buteo*	110	11	10%	1 to 27	Spain	Sanmartin et al. 2004
*C. globifera*	*Accipiter nisus*	35	2	5.7%	1	Spain	Sanmartin et al. 2004
*C. globifera*	*Buteo buteo*	35	3	8.6%	Av.4.3	Italy	Santoro et al. 2012

## Results

Cestodes were found in the intestines of 37 (57%) out of 65 birds at intensities of 1 to 15+ individuals per bird. The prevalence of 57% is considerably higher than currently reported for *Cladotaenia globifera* around the world, when more than 30 individual hosts were investigated (Table 1). The cestodes are fragile and usually appear as pieces of broken strobila, so counts were estimated using the number of scoleces found. This is probably an underestimate considering the very small size of the scoleces and that some may be lost in processing. Using the key to the genera of the Paruterinidae (Georgiev & Kornyushin 1994) the specimens were placed in the genus *Cladotaenia*. Examination of the size and shape of the rostellum and hooks, the morphology of the proglottids, and genetic sequences, found no nominal species comparable to the New Zealand specimens, and they were adjudged to represent a species new to science, which is described below.

**CESTODA Rudolphi, 1808**

**Cyclophyllidea van Beneden in Braun, 1900**

**Paruterinidae Fuhrmann, 1907**

***Cladotaenia*** **Cohn, 1901**

## *Cladotaenia anomala* n. sp. (Figures 1, 2 and 3)

**Figure 1:**
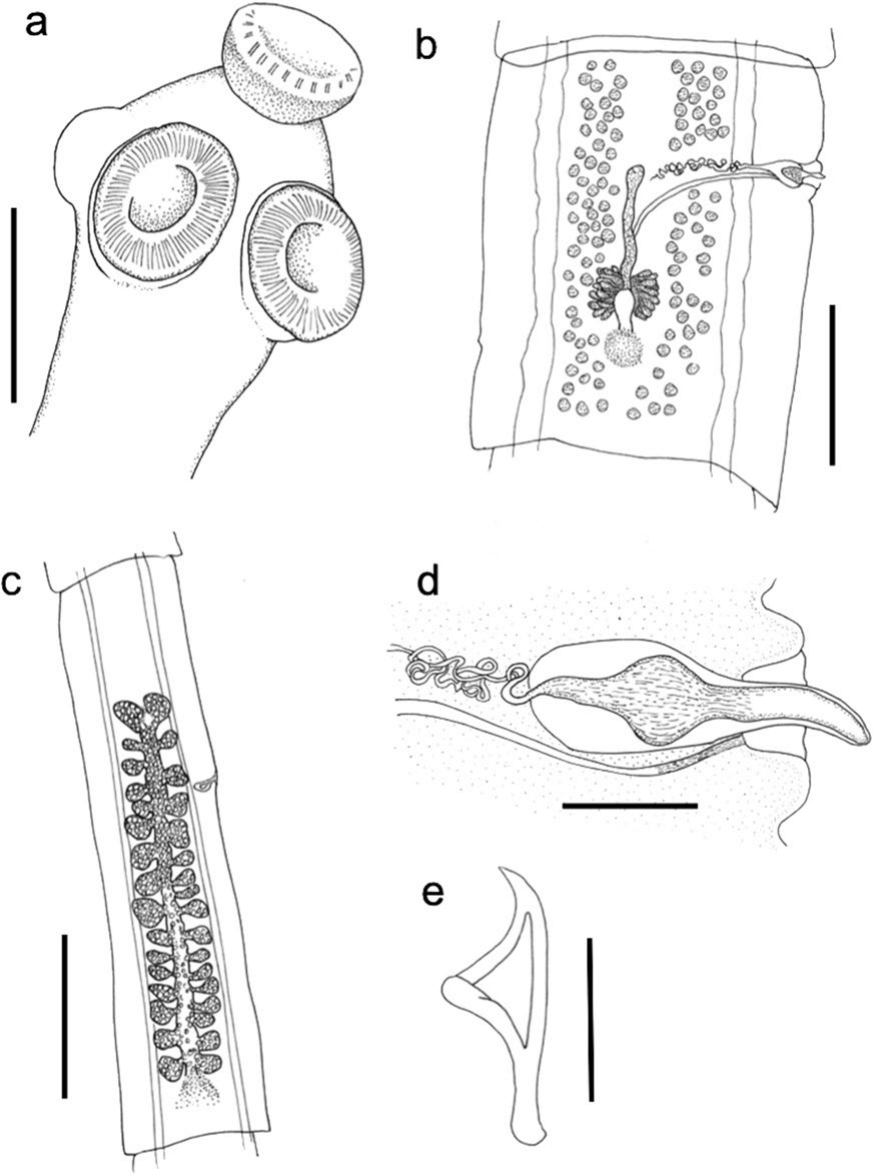
Line drawings of *Cladotaenia anomala* **n. sp.** a) scolex; scars on rostellum mark position of missing hooks, b) Mature proglottid, c) gravid proglottid, d) close-up of genital atrium, e) representative hook. Scale bars: a) 100µm, b) 500µm, c) 1mm, d) 100µm, e) 10µm.

**Figure 2:**
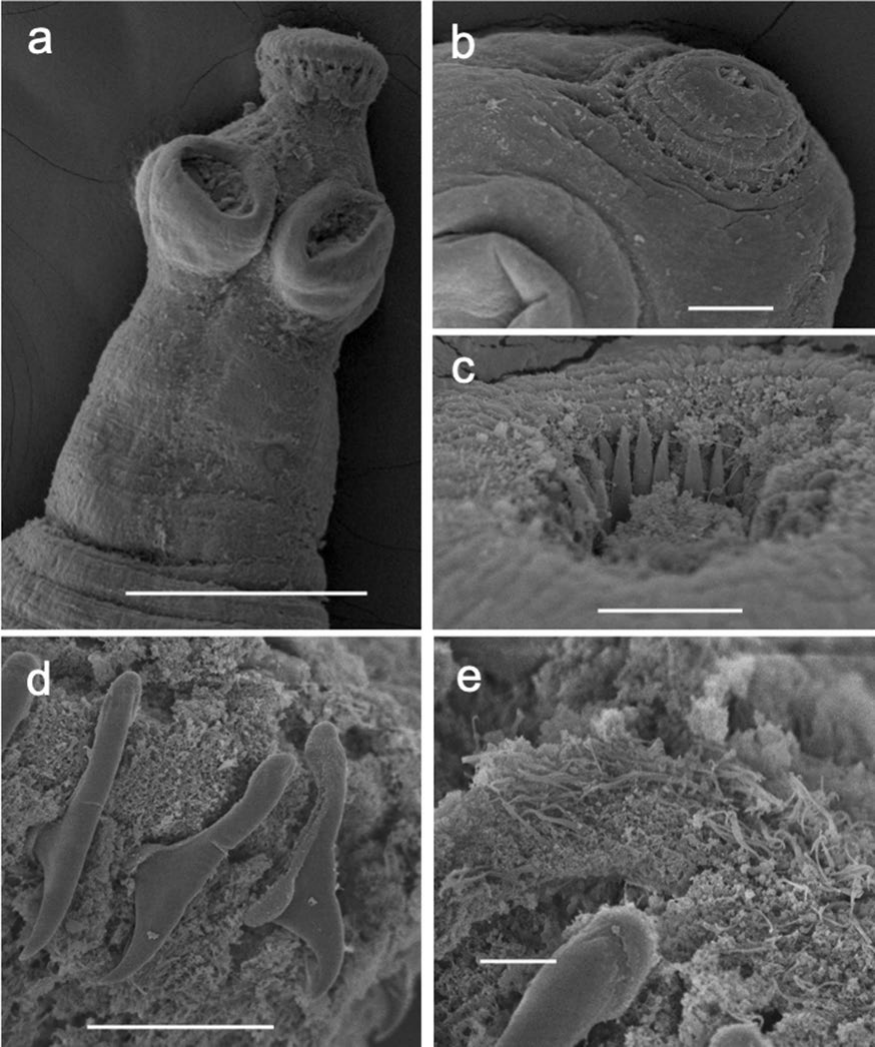
Scanning electron micrographs of *Cladotaenia anomala* **n. sp.** a) scolex and neck, showing suckers and rostellum with hook holes, b) hook holes on partially inverted rostellum on a second specimen, c) hooks in situ in inverted rostellum, d) partially attached hooks showing characteristic shape and epiphyseal thickening, e) capilliform microtriches on rostellar surface. Scale bars: a) 100µm, b) 20µm, c) & d) 10µm, e) 2µm.

**Figure 3:**
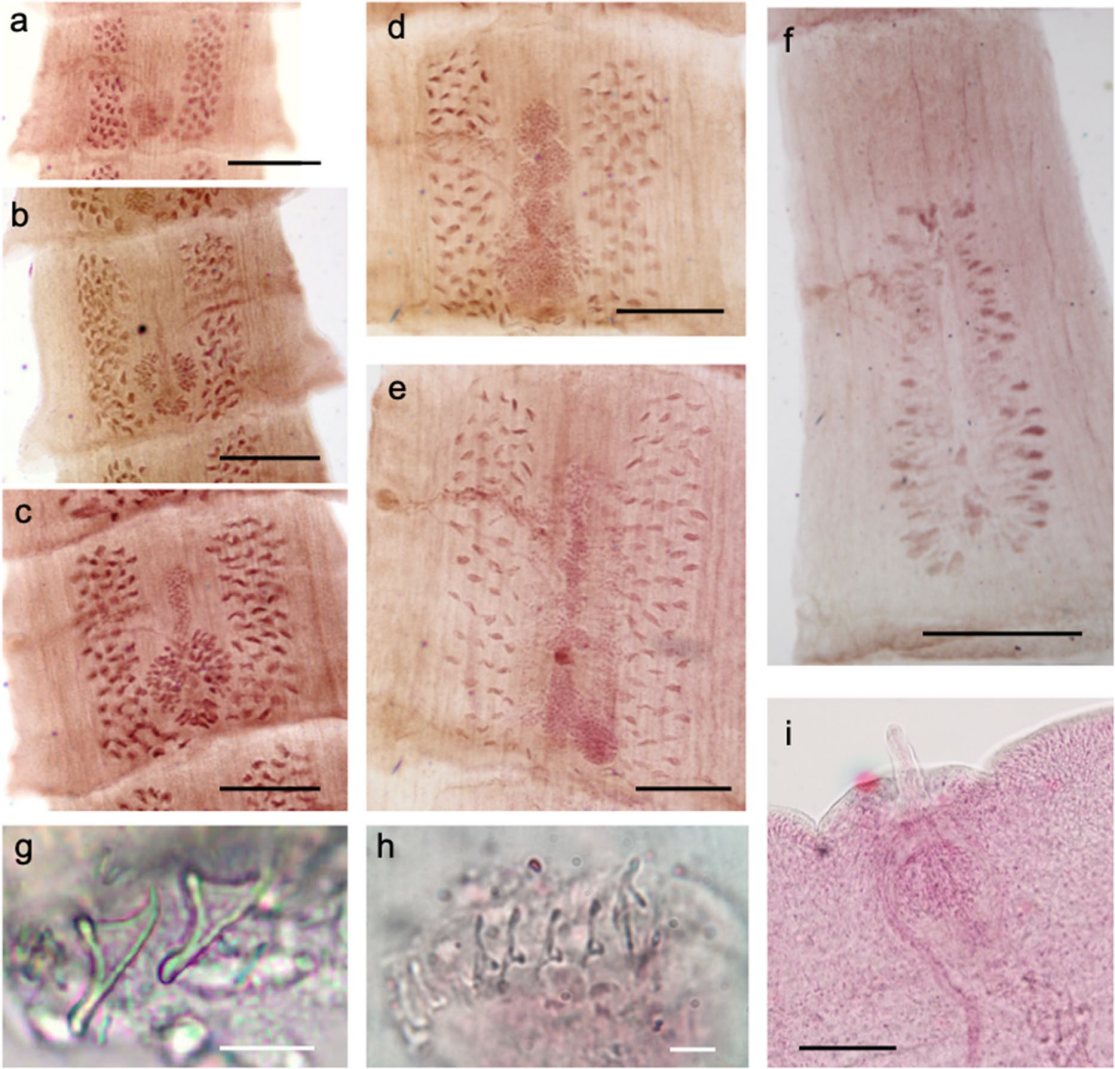
Photomicrographs of *Cladotaenia anomala* **n. sp.** a–f) Proglottids at advancing stages of maturity. a) pre-mature proglottid, b) uterus starting to mature, seen as a thin line, already reaching above level of genital pore, c) uterus begins to swell, d) uterus begins to branch, e) uterus branches becoming differentiated, ovary less dominant, f) uterine branches complete but not yet filling proglottid. g) two adjacent hooks, h) squash preparation showing single hook array on one side of rostellum, i) close-up of genital pore with everted cirrus. Scale bars: a–f) 500µm, g) & h) 10µm, i) 100µm.

*General* [Based on one entire specimen, many partial specimens and 16 scoleces]: Complete specimen with c.300 craspedote proglottids, posterior of which fully gravid; largest entire strobila length 34.4cm, maximum width 2800. Scolex (Fig. 1a, 2a) diameter 138–208 (177). Suckers, slightly cup-shaped, unarmed; 71–95 (81) long x 59–86 (70) wide. Muscular rostellum 35–51 (43) long, when everted, with constriction below the rostellar disc; rostellar disc 67–85 (76) diameter. Rostellar disc bearing capilliform microtriches (Fig. 2e). Rostellar hooks taenioid, with curved blade and epiphyseal thickenings of handle and guard; 20 in single circle (Fig. 1e, 2c, d, 3g, h). Hooks 16–19 total length, width to tip of guard 7.9–8.0, blade 8.7–8.8, handle 12.8–13.3, ratio of hook width to length 1:2.4. Proglottids wider than long, or square, up to point where uterus begins to develop branches, when they become longer than wide. Genital primordia first appear in proglottids number 120–140. Mature proglottids 1300–1800 long x 2200–2800 wide; gravid proglottids ~3.5 times longer than wide, 3500–4325 x 1000–1300 (Fig. 3a – f). Genital pores irregularly alternate; open marginally about one–third length of proglottid from anterior. Genital ducts between osmoregulatory canals. Paruterine organ not strongly demarcated.

Testes 100–108; 56–57 aporal, 17–19 poral anterior to genital ducts, 28–32 poral posterior to genital ducts; subround, 29–33 in diameter, in 2 parallel longitudinal fields between excretory canals, which reach the same level anteriorly, with one or very few testes connecting posterior to vitellarium in mature proglottids (Fig. 1b, 3e). Cirrus sac small, round, not crossing osmoregulatory canals; 70–81 long, 103 wide (Fig. 1d, 3i). Cirrus unarmed, slightly tapering distally, approximately 100 long from exit of cirrus sac when everted, and 30 wide at base (Fig. 3i). Vas deferens, loosely looped upon itself several times, resolves into paired branches at median line of segment; branches enter testicular fields approximately level with genital pore.

Vagina opens in genital atrium posterior to cirrus; of uniform diameter 5–10 wide, wavy at poral end, then describing a smooth curve to proximal end where it passes between lobes of ovary and under posterior portion of uterus (Fig. 1b, d, 3i). No internal or external seminal vesicle. Ovary two-winged, lobed; 136–157 length x 219–234 total width. Vitellarium compact, oval; 73–87 long x 95–115 wide, situated posterior to ovary. Mehlis’ gland lateral oval, anterior to and contiguous with vitellarium, 40 length x 67 width (Fig. 3a – d). Uterus of mature segment slender, becoming club–shaped, extends to anterior of genital pore from early stage of development (Fig. 3b–e). Gravid uterus with elongate central stem each side of which occur 23–27 irregular branches which increase in size with maturity (Fig. 1c, 3f). Eggs subround, 13–15 diameter.

*Type host.* Australasian harrier *Circus approximans* Peale (Accipitriformes: Accipitridae)

*Type locality.* Taieri Plain, Otago 45°53’S, 170°12’E

*Other localities.* East Otago (Dunback, Blueskin Bay, Palmerston, Waikouaiti, Waitati), Dunedin City (Dunedin, Highcliffe, Maungatua, Middlemarch, Saddle Hill, Waldronville), Clutha District (Berwick, Waihola), Canterbury (Darfield, Geraldine, Hinds, Levels Valley, Temuka, Timaru, Twizel), Central Otago (Cromwell, Alexandra).

*Site of infection.* Intestine*.*

*Prevalence and intensity.* In 37 out of 65 birds (57%); intensity 1 to 15 or more (based on number of scoleces).

*Specimens deposited.* Holotype W.003961 (har18, Taieri Plain, 3x slides of strobila); Paratypes W.003962 (har30, Palmerston, 1x slide of strobila pieces and 2x scoleces), W.003963 (har35, Cromwell, 1x slide of 8 scoleces) Museum of New Zealand, Te Papa Tongarewa, Wellington, NZ.

Voucher material. Hologenophores, W.003964 (har30, Palmerston), W.003965 (har43 Geraldine), Museum of New Zealand, Te Papa Tongarewa, Wellington, NZ.

*Other material examined.* Natural History Museum, London 1980.8.27.23–27, three slides consisting of specimens of *Cladotaenia circi* from “swamp harrier” in “New Hebrides” deposited by I.L. Owen.

*Representative DNA sequences*. GenBank Accession 28S: OR844549–OR844554, *cox*1: OR858640–858648.

*Zoobank reference.* urn:lsid:zoobank.org:act:7628CD8A-7119-4592-8620-0C15FFB06D82

*Etymology*. The species name, “*anomala*” refers to the unusual rostellar hook formation. The name is an adjective agreeing in gender with the (feminine) generic name.

### *Remarks*

This paruterinid cestode exhibits a number of characters that define it as a species of *Cladotaenia*: small rostellum, taenioid hooks with epiphyseal thickenings on guard and handle, craspedote proglottids longer than wide when gravid, genital pores irregularly alternating, testes in two longitudinal and lateral fields with minimal connection posteriorly, and reaching the same level anteriorly, small round cirrus sac not crossing osmoregulatory canals, unarmed cirrus, compact oval vitellarium near posterior proglottid margin, two-winged ovary, paruterine organ and uterus with median stem and lateral branches (Georgiev & Kornyushin, 1994). If the synonymisations and reallocations of Freeman (1959) are taken into account there are 12 species of *Cladotaenia* currently valid. The specimens from *C. approximans* were compared to descriptions of all other species.

The new species is distinguished from all other species of *Cladotaenia* by its lack of a second circle of rostellar hooks. As many specimens as possible were examined; as cleared, temporary squashed mounts, as stained and cleared permanent mounts, and as SEM photographs. Hooks are nearly always totally or partially lost, and when present, never seen everted *in situ*. However, the combined evidence confirmed that on none of the specimens was there any indication of a second row of hooks, nor of hook holes, nor of a difference in size between any hooks. Nonetheless, in every other character of the scolex and proglottids, these specimens clearly conform to the diagnosis of the genus *Cladotaenia*. We have to conclude therefore that this is an aberrant species of *Cladotaenia*. This single main difference does not seem sufficient to erect a new genus for the specimens, so we have chosen to include them in *Cladotaenia*. The molecular evidence from the *cox1* gene, which illustrates a close affinity with *Cladotaenia globifera,* also supports this conclusion (see genetic results below).

If the hook circles are disregarded, *C. anomala* **n. sp.** exhibits differences in diagnostic characters from all other species of *Cladotaenia* (Table 2). *Cladotaenia anomala* **n. sp.** is closest morphologically to *C. circi* Yamaguti, 1935, differing mainly in the number of uterine pouches (7–10 as opposed to 23–27 in *C. anomala*), the shape of mature proglottids (wider than long in *C. circi* (Yamaguti 1935) but longer than wide in *C. anomala*) and the larger eggs (18-21 x 15-20 as opposed to 13-15 diameter in *C. anomala*). Despite the genetic closeness with *C. globifera* (see below), morphologically the two species are well separated; *C. globifera* has larger suckers (162 x 97–125 as opposed to 71–95 x 59–86 in *C. anomala*), fewer testes (60–81 as opposed to 100–108 in *C. anomala*) and larger eggs (32 x 34 as opposed to 13–15 diameter in *C. anomala*) (Freeman 1959).

**Table 2:**
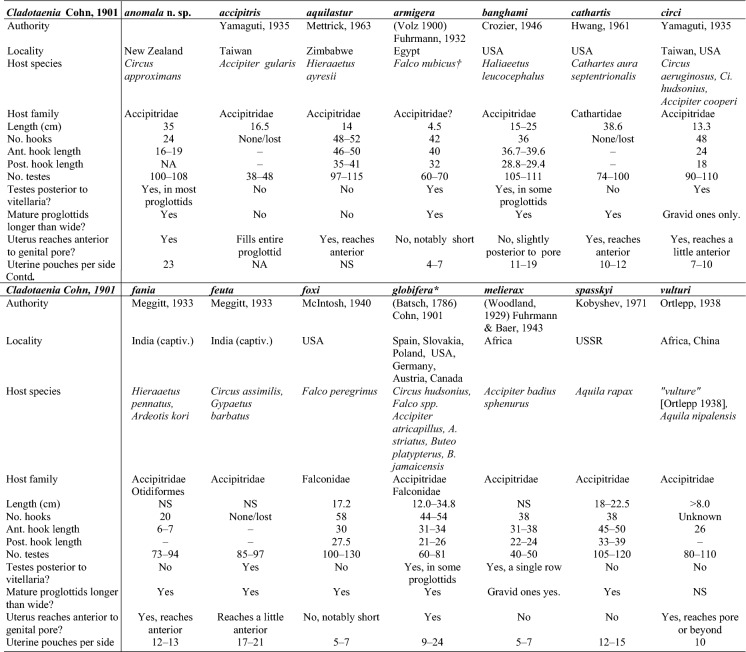
Morpholoigcal comparison of *Cladotaenia* species. Data taken from original descriptions. NS = not stated , – = not applicable or unknown, †Host possibly *Torgos tracheliotos nubicus,* *data taken from Freeman, 1959

### Genetic results

Sequences of the partial 28S gene were obtained for six specimens and all were identical, ranging from 451 to 935 bp in length. A BLASTn search (NCBI) returned the closest available sequence as *Anoplotaenia dasyuri* (81.8% match) from a Tasmanian devil (*Sarcophilus harrisii*), accession MZ618884 (Barton et al. 2021). Although the monotypic genus *Anoplotaenia* Beddard, 1911 is currently considered *incertae sedis* within the Cyclophyllidea (Caira & Jensen, 2017), Barton et al. (2021) using genetic data found *A. dasyuri* to have closest affinity with members of family Paruterinidae, especially *Cladotaenia*. Secondary matches were made with paruterinids, *Anonchotaenia macrocephala* (77.5% ) (KF685922) and *Anonchotaenia cf. brasiliensis* (80.27%) (KF685923) (Phillips et al. 2014), although coverage was not 100%. No other 28S sequences of *Cladotaenia* were available on GenBank, precluding a useful phylogenetic hypothesis.

Sequences of the *cox*1 gene were obtained for nine specimens infecting six harriers, which ranged from 374 to 379 bp in length. Four unique haplotypes were identified (See Table 3) and the uncorrected pairwise genetic divergence between the four haplotypes ranged from 0.26–1.33% with an average divergence of 0.80%. Over the 376 bp alignment, 6 bp showed polymorphisms, including changes at bp positions 10, 52, 93, 132, 240 and 285. Based on a BLASTn search, the closest representative is *Cladotaenia globifera* from the liver of a striped field mouse (*Apodemus afrarius*) in Poland (accession MN514029, Bajer et al. (2020)) which matched 97.06–98.14% (1.86–2.94% divergence) with the New Zealand sequences. A sequence attributed to *C. vulturi* (NC_032067, Guo 2016) is considerably distant from other *Cladotaenia* sequences for reasons that are unclear. *Cladotaenia globifera* and *C. vulturi* exhibited 14.6% genetic divergence between them, which is almost as high as the overall mean genetic divergence (17.49%) exhibited between currently available genera in Paruterinidae (i.e. *Anonchotaenia*, *Cladotaenia* and *Dictyterina*).

**Table 3:** Data regarding representatives of *Cladotaenia anomala* **n. sp.** ex Australasian harriers chosen for DNA sequencing with associated haplotypes and GenBank Accession numbers.

Specimen ID	Locality	28S	*cox*1	
		GenBank Accession	Haplotype	GenBank Accession
Har18ces1	Taeiri Plain	OR844549	Hap1	OR858640
Har30ces4	Palmerston	OR844550	Hap1	OR858641
Har30ces5	Palmerston	OR844551	Hap1	OR858642
Har40ces2	Dunback		Hap2	OR858643
Har40ces3	Dunback	OR844552	Hap2	OR858644
Har41ces1	Dunedin	OR844553	Hap3	OR858645
Har43ces1A	Geraldine		Hap4	OR858646
Har45ces1	Hinds		Hap4	OR858647
Har45ces3	Hinds	OR844554	Hap4	OR858648

The 28S and *cox*1 sequences presented here are made available to contribute to future hypotheses regarding phylogenetic history and interrelationships within Paruterinidae as more representatives of the family become available (Table 3).

## Discussion

Species of *Cladotaenia* occur almost exclusively in birds of prey belonging to Falconidae and Accipitridae (one species, *C. cathartis* Hwang, 1961 occurs in a new world vulture, Cathartidae), with *C. circi* (ex *Ci. aeruginosus* (L.)), *C. feuta* Meggitt 1933 (ex *Ci. assimilis* Jardine & Selby), *C. globifera* (ex *Ci. hudsonius* (L.) and *Ci. pygargus* (L.)) recorded from *Circus* species (Freeman 1959; Jones 1930; Komorová et al. 2017; Meggitt 1933; Yamaguti 1935). No species of *Cladotaenia* has been recorded in New Zealand previously, and this is the first report of any cestode occurring in the Australasian harrier, *Ci. approximans* in New Zealand. Mawson et al. (1986) found an unnamed specimen of *Cladotaenia* ex *Ci. approximans* from Victoria in the Australian Helminth Collection (now South Australia Museum), but no morphological details were given, and specimens of *C. circi* ex *Ci. approximans* in Vanuatu are in the Natural History Museum, London and were examined by us for this study. It has been over 50 years since a new species of *Cladotaenia* was last described, although species have been included in a few molecular studies (Bajer et al. 2020; Barton et al. 2021; Guo 2016), and records of described and undescribed species occur in various helminth assemblage studies (González-Acuña & Lohse 2011; Komorová et al. 2017; Krone 2000; Sanmartín et al. 2004; Santoro et al. 2012).

The Australasian harrier is the only accipitrid raptor found in New Zealand. The only other diurnal raptor now extant is the New Zealand falcon, a rarely-seen bird of open country, with Threatened status (Robertson et al. 2021). We have examined four deceased falcons and found no cestodes of any kind in the gastrointestinal tract. We therefore infer that *C. anomala* **n. sp.** is host-specific to the harrier. The intermediate hosts of *Cladotaenia* spp. are known to be small rodents and insectivorous mammals (Georgiev & Kornyushin 1994; Bajer et al. 2020). Apart from two bat species, there are no native mammals in New Zealand. The Australasian harrier probably became established following the habitat disturbances associated with humans in the last 800 years (Holdaway & Worthy 1997), and, with no native mammals present at the time, the cestode clearly did not become established in New Zealand with the first arrival of its definitive host. There are no records in the literature of *Cladotaenia* infection in any small mammals of New Zealand, but the only reasonable contenders for potential intermediate hosts are the introduced house mouse *Mus musculus* L., rats *Rattus rattus* (L.), *R. norvegicus* (Berkenhout) and *R. exultans* (Peale), and the hedgehog *Erinaceus europaeus* L.

Mice were introduced accidentally to New Zealand, probably on numerous occasions, since the arrival of Europeans in the last 200 years (Searle et al. 2009). Interestingly, it has been demonstrated using genetic data that mouse samples from the southern South Island belong to the subspecies *M. m. castaneus*, which originates from southeast Asia (Searle et al. 2009). Did the mice that carried *Cladotaenia* merocercoid larvae arrive from Asia? Of three *Cladotaenia* species reported from that region, two are *C. circi* and *C. accipitris* (=*Paracladotaenia accipitris*), both described by Yamaguti (1935) from Taiwan, the former in *Ci. aeruginosus.* It seems possible that one or both of these species may be closely related to the New Zealand species, but until genetic data are available, their relationship with *C. anomala* **n. sp.** remains speculative.

Three species of rat are found in New Zealand. Polynesian rats *Rattus exultans* were transported with the earliest human settlers about 1000 years ago and were once widespread throughout the country, but are now restricted to limited parts of Fiordland, Southland and south Westland (Wilmshurst & Ruscoe 2021). They are therefore unlikely hosts for *C. anomala* **n. sp.** since the cestode maintains a much wider distribution than this rat species. The other two species, the Norway rat *Rattus norvegicus* and the ship rat *Rattus rattus* arrived with early European ships, in the late 18^th^ century and the late 19^th^ century respectively (King & Veale 2022). Interestingly, *R. norvegicus* populations from South Island show a high proportion of South-East Asian ancestry, in common with that of house mice (Puckett et al. 2016), supporting the hypothesis of a potential Asian origin. Mice make up a small fraction of the diet of harriers, but rats have rarely been reported in regurgitates or stomach contents (Baker-Gabb 1981; Carroll 1968; Pierce and Maloney 1989; Redhead 1969).

While roadkill hedgehogs undoubtedly make up a significant fraction of the harrier’s diet (Baker-Gabb 1981), no *Cladotaenia* species has been reported from a hedgehog in New Zealand, this despite a large sample of these insectivores having been examined for a study on acanthocephalan parasites (Skuballa et al. 2010). Hedgehogs have been found infected with *C. globifera* in the Azores archipelago, Portugal (Casanova et al. 1996). If *C. anomala* **n. sp.** indeed infects hedgehogs in New Zealand it arrived as recently as 1870 when these mammals were first introduced from Great Britain. No species of *Cladotaenia* has been reported from any raptors in Britain, so the origin of the New Zealand species for the moment remains a mystery.

Based on our findings it appears that New Zealand harriers (at least in the South Island of New Zealand) host only one cestode species, *Cladotaenia anomala* **n. sp.** The prevalence of this cestode was relatively high compared to other harrier-*Cladotaenia* associations known in the literature from other parts of the world (see Table 1). Komorová et al. (2017) investigated cestodes infecting a range of birds of prey and reported some accipitriform raptors hosted up to six cestode species. Some *Cladotaenia* species were also found to exhibit relatively strict host specificity, such as *C. globifera* which infects three raptor species in Slovakia. The concentration of *C. anomala* **n. sp.** in New Zealand harriers may well reflect the recent arrival of intermediate host species and potential lack of competition by other cestodes.

This new species provides a potentially interesting example of a parasite’s colonisation of new host species and geographical areas, since the dates or arrival and origin of its potential intermediate host species are known. Several study routes could be followed to elucidate this pattern: *Circus approximans* should be examined in Australia for the presence of *C. anomala*, introduced mammals in New Zealand should be examined for metacestodes to establish the intermediate host, and a more rigorous genetic survey of *Cladotaenia* specimens from different host taxa in other parts of the world would place this species into phylogenetic context.

## Data Availability

Not applicable.

## References

[CR1] Abuladze, K. I. (1958) The characteristics of cestodes of the genus *Cladotaenia* Colin, 1901. *Papers on Helminthology presented to Academician K. I. Skrjabin on his 80th birthday*. Moscow: Izdatelstvo Akademii Nauk [in Russian] S.S.S.E., pp. 28–31.

[CR2] Bajer A, Alsarraf M, Dwużnik D, Mierzejewska EJ, Kołodziej-Sobocińska M, Behnke-Borowczyk J, Banasiak Ł, Grzybek M, Tołkacz K, Kartawik N, Stańczak Ł, Opalińska P, Krokowska-Paluszak M, Górecki G, Alsarraf M, Behnke JM (2020). Rodents as intermediate hosts of cestode parasites of mammalian carnivores and birds of prey in Poland, with the first data on the life-cycle of *Mesocestoides melesi*. Parasites & vectors.

[CR3] Baker-Gabb DJ (1981). The diet of the Australasian harrier (*Circus approximans*) in the Manawatu-Rangitikei sand country, New Zealand. Notornis.

[CR4] Barton DP, Zhu X, Lee V, Shamsi S (2021). The taxonomic position of *Anoplotaenia dasyuri* (Cestoda) as inferred from molecular sequences. Parasitology.

[CR5] Bennett J, Presswell B (2019). Morphology and molecules resolve the identity and life cycle of an eye trematode, *Philophthalmus attenuatus* n. sp. (Trematoda: Philophthalmidae) infecting gulls in New Zealand. Parasitology Research.

[CR6] Bennett J, Presswell B, Poulin R (2023). Tracking life cycles of parasites across a broad taxonomic scale in a marine ecosystem. International Journal for Parasitology.

[CR7] Bowles J, Blair D, Mcmanus D (1992). Genetic variants within the genus *Echinococcus* identified by mitochondrial DNA sequencing. Molecular and biochemical parasitology.

[CR8] Caira, J. N., & Jensen, K. (2017) Planetary biodiversity inventory (2008-2017): Tapeworms from vertebrate bowels of the earth. *University of Kansas Natural History Museum Special Publication,**25*, 1–463. http://hdl.handle.net/1808/24421

[CR9] Carroll ALK (1968). Foods of the harrier. Notornis..

[CR10] Casanova JC, Miquel J, Fons R, Molina X, Feliu C, Mathias MDL, Torres J, Libois R, Santos-Reis M, Collares-Pereira M, Marchand B (1996). On the helminthfauna of wild mammals (Rodentia, Insectivora and Lagomorpha) in Azores archipelago (Portugal). Vie et Milieu.

[CR11] Debus, S. & Kirwan, G. M. (2020) Swamp Harrier (*Circus approximans*), version 1.0. In del Hoyo, J., Elliott, A., Sargatal, J., Christie, D. A., & de Juana E., (eds.) *Birds of the World*. Cornell Lab of Ornithology, Ithaca, NY, USA. 10.2173/bow.swahar1.01

[CR12] Eakle WL (2008). Relative abundance of Australasian harriers (*Circus approximans*) in New Zealand. Notornis.

[CR13] Freeman, R. S. (1959) On the taxonomy of the genus *Cladotaenia*, the life histories of *C. globifera* (Batsch, 1786) and *C. circi* Yamaguti, 1935, and a note on distinguishing between the plerocercoids of the genera *Paruterina* and *Cladotaenia*. *Canadian journal of zoology**37*(3), 317–340. 10.1139/z59-037

[CR14] Fuhrmann O, Baer JG (1943). Cestodes. Bulletin de la Société Neuchâteloise des Sciences Naturelles.

[CR15] Georgiev, B. B. & Kornyushin, V. V. (1994) Family Paruterinidae Fuhrmann, 1907 (sensu lato). In: Khalil, L.F., Jones, A. & Bray, R. A. (eds.) *Keys to the cestode parasites of vertebrates*. pp. 559–584.

[CR16] González-Acuña D, Lohse E (2011). Parasites of the American kestrel (*Falco sparverius*) in south-central Chile. Journal of Raptor Research.

[CR17] Guo A (2016). The complete mitochondrial genome of the tapeworm *Cladotaenia vulturi* (Cestoda: Paruterinidae): gene arrangement and phylogenetic relationships with other cestodes. Parasites & Vectors.

[CR18] Guo A, Wang L, Zhang S, Zheng Y, Georgiev BB, Luo X, Huang S, Cai X (2019). Mitochondrial genome of *Paruterina candelabraria* (Cestoda: Paruterinidae), with implications for the relationships between the genera *Cladotaenia* and *Paruterina*. Acta tropica.

[CR19] Harper JT, Saunders GW (2001). Molecular systematics of the Florideophyceae (Rhodophyta) using nuclear large and small subunit rDNA sequence data. Journal of Phycology.

[CR20] Holdaway RN, Worthy TH (1997). A reappraisal of the late quaternary fossil vertebrates of Pyramid Valley Swamp, North Canterbury, New Zealand. New Zealand Journal of Zoology.

[CR21] Hwang, J. C. (1961) *Cladotaenia (Paracladotaenia) cathartis* n. sp.(Cestoda: Taeniidae) from the Intestine of the Turkey Buzzard, *Cathartes aura septentrionalis* Wied, 1893. *The Journal of Parasitology,*, *47*(2), 205–207. 10.2307/3275289 13717126

[CR22] Jones, M. (1930) [Note without title. Proceedings of the Helminthological Society of Washington]. *Journal of Parasitology,*, *16*(3), 159.

[CR23] Joyeux, C. H. & Baer, J-G. (1961) Classe des cestodes. In: Grasse, P.P. (ed.). *Traité de Zoologie. Anatomie, Systematique, Biologie, Vol. 4, Fasc. 1. Platyhelminthes, Mesozaires, Acanthocéphales, Nemertiens*. Masson, Paris, pp. 347–560.

[CR24] King, C., & Veale, A. (2022) New light on the introduction of ship-borne commensal rats and mice in Aotearoa New Zealand, 1790s-1830s. *International Review of Environmental History,**8*(2), 75-102. 10.22459/IREH.08.02.2022.05

[CR25] Komorová P, Sitko J, Špakulová M, Hurníková Z, Sałamatin R, Chovancová G (2017). New data on helminth fauna of birds of prey (Falconiformes, Accipitriformes, Strigiformes) in the Slovak Republic. Helminthologia.

[CR26] Králová-Hromadová I, Špakulová M, Horáčková E, Turčeková L, Novobilský A, Beck R, Koudela B, Marinculić A, Rajský D, Pybus M (2001). Sequence analysis of ribosomal and mitochondrial genes of the giant liver fluke *Fascioloides magna* (Trematoda: Fasciolidae): intraspecific variation and differentiation from *Fasciola hepatica*. Journal of Parasitology.

[CR27] Krone O, Lumeij JT, Remple JD, Redig PT, Lierz M, Cooper JE (2000). Endoparasites in free-ranging birds of prey in Germany. Chapter 10. Raptor biomedicine III.

[CR28] Mariaux, J., Tkach, V. V., Vasileva, G. P., Waeschenbach, A., Beveridge, I., Dimitrova, Y. D., Haukisalmi, V., Greiman, S.E., Littlewood, D.T.J., Makarikov, A.A., Phillips, A.J., Razafiarisolo, T., Widmer, V., Georgiev, B. B. (2017) Cyclophyllidea van Beneden in Braun, 1900. In: Caira, J. N. & Jensen, K. (eds.), *Planetary Biodiversity Inventory (2008–2017): Tapeworms from Vertebrate Bowels of the Earth.* University of Kansas, Natural History Museum, Special Publication No. 25, Lawrence, KS, pp. 77–148.

[CR29] Mawson PM, Angel LM, Edmonds SJ (1986). A check list of helminths from Australian birds. Records of the South Australian Museum.

[CR30] Meggitt FJ (1933). Cestodes obtained from animals dying in the Calcutta zoological gardens during 1931. Records of the Zoological Survey of India.

[CR31] Miller, M.A., Pfeiffer, W., Schwartz, T. (2010) Creating the CIPRES Science Gateway for Inference of Large Phylogenetic Trees. In: *Proceedings of the 2010 Gateway Computing Environments Workshop (GCE)*, New Orleans, LA, USA, 14 November 2010; pp. 1–8. 10.1109/GCE.2010.5676129

[CR48] Presswell, B., & Bennett, J. (2023) Description and molecular data for a new acanthocephalan parasite, *Polymorphus circi *n. sp.(Polymorphidae) from the Australasian harrier (*Circus approximans *Peale) in New Zealand. *Systematic Parasitology,*, *100*(6), 725–733. 10.1007/s11230-023-10120-5 10.1007/s11230-023-10120-5 PMC10613132 37874424

[CR49] Presswell, B., & Bennett, J. (2024) Gastrointestinal helminths of the Australasian harrier (*Circus approximans *Peale, 1848) in New Zealand, and description of a new species of nematode, *Procyrnea fraseri *n. sp. (Habronematidae). *Journal of Helminthology,**98*, e6, 1–12. 10.1017/S0022149X23000883 10.1017/S0022149X23000883 38213187

[CR32] Phillips, A. J., Georgiev, B. B., Waeschenbach, A., & Mariaux, J. (2014) Two new and two redescribed species of *Anonchotaenia* (Cestoda: Paruterinidae) from South American birds. *Folia Parasitologica,**61*(5), 441. 10.14411/fp.2014.058 25549500

[CR33] Pierce RJ, Maloney RF (1989). Responses of harriers in the Mackenzie Basin to the abundance of rabbits. Notornis.

[CR34] Puckett EE, Park J, Combs M, Blum MJ, Bryant JE, Caccone A, Costa F, Deinum EE, Esther A, Himsworth CG, Keightley PD (1841). 2016) Global population divergence and admixture of the brown rat (*Rattus norvegicus*. Proceedings of the Royal Society B: biological sciences.

[CR35] Rausch, R. L. (1985) Presidential address. Parasitology: retrospect and prospect. *Journal of Parasitology**71*(2), 139–151. 3889262

[CR36] Redhead RE (1969). Some aspects of the feeding of the harrier. Notornis.

[CR37] Robertson, H. A., Baird, K. A., Elliott, G., Hitchmough, R., McArthur, N., Makan, T., Miskelly, C., O'Donnell, C. F., Sagar, P. M., Scofield, R. P. and Michel, P. (2021) *Conservation status of birds in Aotearoa New Zealand, 2021.* Department of Conservation, Te Papa Atawhai, Wellington, New Zealand. 43pp.

[CR38] Sadleir RM, Linklater WL (2016). Annual and seasonal patterns in wildlife road-kill and their relationship with traffic density. New Zealand journal of zoology.

[CR39] Sanmartín ML, Alvarez F, Barreiro G, Leiro J (2004). Helminth fauna of Falconiform and Strigiform birds of prey in Galicia, Northwest Spain. Parasitology Research.

[CR40] Santoro M, Kinsella JM, Galiero G, Uberti BD, Aznar FJ (2012). Helminth community structure in birds of prey (Accipitriformes and Falconiformes) in southern Italy. Journal of Parasitology.

[CR41] Schmelz O (1941). Quelques cestodes nouveaux d’oiseaux d’Asie. Revue Suisse de Zoologie.

[CR42] Schmidt GD (1986). CRC Handbook of tapeworm identification.

[CR43] Searle JB, Jamieson PM, Gündüz I, Stevens MI, Jones EP, Gemmill CE, King CM (2009). The diverse origins of New Zealand house mice. Proceedings of the Royal Society B: Biological Sciences.

[CR44] Skuballa J, Taraschewski H, Petney TN, Pfäffle M, Smales LR (2010). The avian acanthocephalan *Plagiorhynchus cylindraceus* (Palaeacanthocephala) parasitizing the European hedgehog (*Erinaceus europaeus*) in Europe and New Zealand. Parasitology research.

[CR45] Wilmshurst JM, Ruscoe WA (2021) *Rattus exulans*. In *The handbook of New Zealand mammals*. 3rd edn. (C. M. King and D. M. Forsyth, Editors) Family Muridae, pp. 161–240. CSIRO Publishing, Melbourne. http://ebookcentral.proquest.com/lib/otago/detail.action?docID=6471514.

[CR46] Yamaguti, S. (1935) Studies on the helminth fauna of Japan. Part 6. Cestodes of birds, I. *Japanese Journal of Zoology**6*(2), 184-232

[CR47] Yamaguti S (1959). Systema Helminthum II.

